# Modulating electronic structure of metal-organic frameworks by introducing atomically dispersed Ru for efficient hydrogen evolution

**DOI:** 10.1038/s41467-021-21595-5

**Published:** 2021-03-01

**Authors:** Yamei Sun, Ziqian Xue, Qinglin Liu, Yaling Jia, Yinle Li, Kang Liu, Yiyang Lin, Min Liu, Guangqin Li, Cheng-Yong Su

**Affiliations:** 1 MOE Laboratory of Bioinorganic and Synthetic Chemistry, Lehn Institute of Functional Materials, School of Chemistry, Sun Yat-Sen University, Guangzhou, China; 2 School of Physics and Electronics, Central South University, Changsha, Hunan China

**Keywords:** Metal-organic frameworks, Electrocatalysis, Nanoscale materials

## Abstract

Developing high-performance electrocatalysts toward hydrogen evolution reaction is important for clean and sustainable hydrogen energy, yet still challenging. Herein, we report a single-atom strategy to construct excellent metal-organic frameworks (MOFs) hydrogen evolution reaction electrocatalyst (NiRu_0.13_-BDC) by introducing atomically dispersed Ru. Significantly, the obtained NiRu_0.13_-BDC exhibits outstanding hydrogen evolution activity in all pH, especially with a low overpotential of 36 mV at a current density of 10 mA cm^−2^ in 1 M phosphate buffered saline solution, which is comparable to commercial Pt/C. X-ray absorption fine structures and the density functional theory calculations reveal that introducing Ru single-atom can modulate electronic structure of metal center in the MOF, leading to the optimization of binding strength for H_2_O and H*, and the enhancement of HER performance. This work establishes single-atom strategy as an efficient approach to modulate electronic structure of MOFs for catalyst design.

## Abstract (web summary)

Developing high-performance, neutral-media H_2_-evolution electrocatalysts is important for clean and sustainable hydrogen energy, yet rare, expensive elements are most active. Here, authors show that metal-organic frameworks modified with single ruthenium atoms as high-performances catalysts.

## Introduction

The increasing consumption of fossil fuels and deterioration of living environment drive people to explore environmental friendly and sustainable energy sources as alternatives for the traditional fossil fuels^1–4^. Among them, hydrogen is considered as the most promising substitute because of its high gravimetric energy density as well as zero CO_2_ emission^5–8^. Recently, electrochemical water splitting, generally including two half-reactions, hydrogen evolution reaction (HER) and oxygen evolution reaction (OER), has aroused increasing interests^9–12^. HER, producing low-cost and high purity hydrogen gas, is the hot spot in energy-conversion technologies and arouses increasing interests. Up to now, Pt is recognized as the high-performance electrocatalyst due to its fast dynamics and low overpotential^13–16^. Despite of the high efficiency, the high cost and scarcity impede its large-scale application and drive people to pursue more cheap and efficient electrocatalysts^17–21^.

Metal-organic frameworks (MOFs) are a class of emerging porous crystalline materials^22–25^ composed of variety organic ligands and metal centers with various applications, such as water splitting^26–29^, gas storage^30–32^ and metal–air batteries^33–37^. Benefitting from flexible tunability and well-defined structure of MOFs, their performance can be optimized using fundamental molecular chemistry principles^38^. This makes MOFs as promising model catalysts for investigating the design of catalysts at the molecular level. Though, some approaches including metal node engineering^39^, missing-linker MOF^40^ and lattice-strained MOF^41^ have been reported to design advanced OER electrocatalysts. Developing efficient strategies to regulate electrocatalytic performance of MOFs for HER is challenging.

Currently, single-atom catalysts (SACs) have intrigued new interests in heterogenous electrocatalysis because of their outstanding activity and maximum atom utilization efficiency^42,43^. The impressive catalytic activity of SACs greatly results from the distinctive electronic structure of single metal atoms and the interaction between single metal atoms and supports^44–47^. So, beyond serving as active sites, incorporating single-atom metals into catalysts can also lead to the local electronic structure modulation of initial catalysts^48^, owning to the electronic interaction between them. Moreover, the incorporation of atomically dispersed single-atom can preserve the structural feature of original materials. All of these aspects provide promising opportunity to introduce single-atom to enhance the electrocatalytic performance of MOFs.

Herein, we propose a single-atom strategy to tailor HER performance of the MOF Ni-BDC (Ni_2_(OH)_2_(C_8_H_4_O_4_)^49,50^, H_2_BDC: terephthalic acid) by introducing atomically dispersed Ru (named as NiRu_0.13_-BDC). Remarkably, the as-synthesized catalyst performs enhanced activity toward HER in all pH. The optimized NiRu_0.13_-BDC catalyst exhibits high HER performance with a low overpotential of 36 mV at 10 mA cm^−2^ and a Tafel slope of 32 mV dec^−1^, which are much improved compared with pure Ni-BDC in 1 M phosphate buffered saline (PBS) solution. More importantly, combining with calculation results, the electronic structure of Ni can be regulated by the construction of Ru single-atom into Ni-BDC, thus optimizing the adsorption strength for H_2_O and H* and contributing to the enhanced performance.

## Results

### Synthesis and characterization of NiRu_0.13_-BDC

**Fig. 1:**
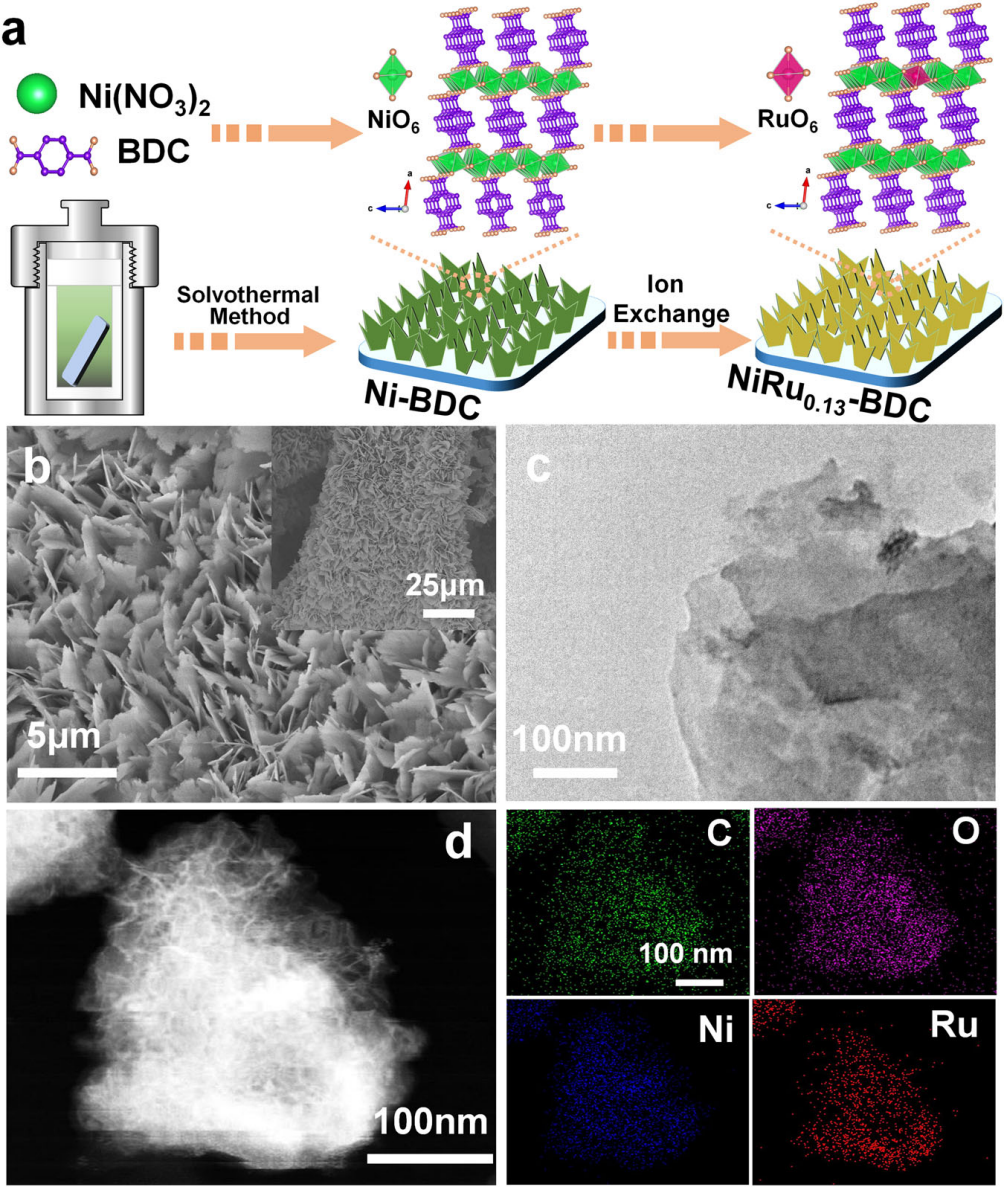
Schematic of sample preparation and physical characterization of NiRu_0.13_-BDC. **a** Schematic illustration for the preparation of NiRu_0.13_-BDC catalyst. **b** SEM; **c** TEM images of NiRu_0.13_-BDC. **d** HAADF-STEM image and the corresponding STEM-EDS mappings of NiRu_0.13_-BDC.

First, a pristine MOF nanosheet array supported on Ni foam was prepared by a hydrothermal method. Furthermore, the structure of initial MOF material was introduced as “Ni-BDC” with layered-pillared structure constructed by the coordinated octahedrally divalent Ni and terephthalic acid (H_2_BDC), where the terephthalates are coordinated and pillared directly to the Ni hydroxide layers and form a three-dimensional framework (Supplementary Fig. 1)^50^. Ru single-atom catalyst supported on Ni-BDC growing on nickel foam was synthesized by replacing part of Ni atoms through an ion-exchange method (Fig. 1a, Supplementary Fig. 1b and details in the Method section). Through varying the amount of RuCl_3_, a series of catalysts with different loading amount of Ru were synthesized. The content of Ru was determined by inductively coupled plasma mass spectrometry (ICP-MS) (Supplementary Table 1). The structures of these materials were firstly studied by powder X-ray diffraction (XRD). As revealed in Supplementary Fig. 4, NiRu_0.09_-BDC and NiRu_0.13_-BDC have the similar diffraction patterns as Ni-BDC, the diffraction peak appeared at 8.9 was identified to the characteristic (2,0,0) facet of the Ni-BDC^49,50^, indicating the similar crystal structure. When the loading amount is higher than 13%, the structure of the MOF was destroyed, as shown in Supplementary Figs. 2 and 4. To further certify the structure of NiRu_0.13_-BDC, NiRu_0.13_-BDC powder sample was also synthesized via the same method excepting for the addition of nickel foam. As shown in Supplementary Fig. 5, the NiRu_0.13_-BDC powder exhibited the similar diffraction peaks as Ni-BDC.

The morphologies and microstructures of these catalysts were deeply investigated by scanning electron microscope (SEM) and transmission electron microscope (TEM). As disclosed in Fig. 1b, c and Supplementary Fig. 2, NiRu_0.13_-BDC, NiRu_0.09_-BDC, and Ni-BDC showed uniform nanosheets morphology assembling on nickel foam. High-resolution TEM images in Supplementary Fig. 3 revealed the lattice fringe spacing of 1.04 nm, corresponding to the (200) plane of Ni-BDC. After introducing Ru, the lattice fringe spacing kept unchanged and no obvious nanoparticles exhibited. When the loading amount of Ru increased to 21%, the structure of NiRu_0.21_-BDC was completely destroyed, only aggregated bulk formed. The energy dispersive spectroscopy (EDS) of NiRu_0.13_-BDC in Fig. 1d further demonstrated the elements of C, O, Ni, and Ru distribute uniformly. In addition, ICP-MS analysis was also collected to determine the elements content, showing coexistence of Ni and Ru with a molar ratio of 0.13 (Ru/Ni), further confirming the successful doping of Ru.

**Fig. 2:**
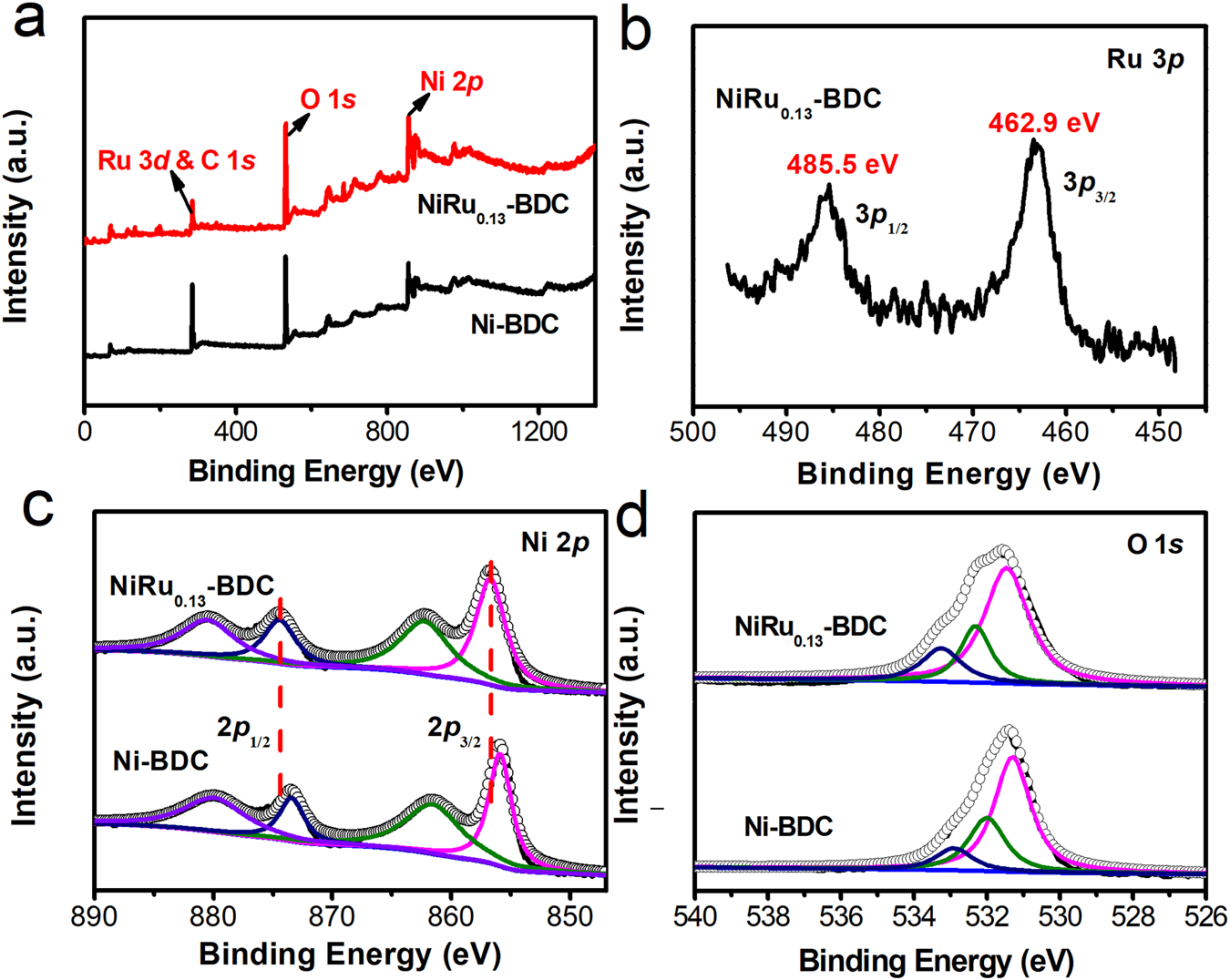
X-ray photoelectron spectroscopic studies. **a** full range XPS spectra, **b** Ru 3*p*, **c** Ni 2*p* and **d** O 1*s* spectra of Ni-BDC and NiRu_0.13_-BDC.

To further investigate the chemical composition and electronic structure of the NiRu_0.13_-BDC, X-ray photoelectron spectroscopy (XPS) was conducted. From Fig. 2a and Supplementary Fig. 6, the XPS spectra of the NiRu_0.13_-BDC demonstrated the coexistence of Ni, Ru, O, and C elements. Compared with Ni-BDC, after introducing Ru atoms, the Ru 3*p* spectra (Fig. 2b) can be clearly detected in NiRu_0.13_-BDC, further proving the successful doping of Ru. In addition, the Ru 3*p* peaks located at 462.9 and 485.5 eV were assigned to Ru^3+^ 3*p*_3/2_ and Ru^3+^ 3*p*_1/2_, conforming the oxided state of Ru rather than metallic^51–56^. Meanwhile, there is a pair of peaks with an energy of 281.9 and 286.4 eV, which can be assigned to Ru 3*d*_5/2_ and Ru 3*d*_3/2_ in the C 1*s* and Ru 3*d* spectra (Supplementary Fig. 6) in comparision with Ni-BDC^51–56^, indicating the valance state of Ru^3+^ and the successful incorporation of Ru single-atom into Ni-BDC. The remaining three peaks can be contributed to C=C bond at 284.8 eV, C–O bond at 285.8 eV and O–C=O bond at 288.8 eV^57^, in conformity with that of Ni-BDC. For NiRu_0.09_-BDC (Supplementary Fig. 6b), the Ru 3*p* peaks with binding energy of 463.4 and 485.8 eV were also result from the oxidized Ru. While, from Ru 3*p* of NiRu_0.21_-BDC, the peaks located at 461.7 and 484.3 eV for Ru 3*p*_3/2_ and 3*p*_1/2_ are assigned to metallic Ru, indicating the formation of Ru nanoparticles^51–56^. The Ni 2*p* spectra (Fig. 2c) of Ni-BDC show two characteristic peaks at 855.9 and 873.2 eV, identified as Ni 2*p*_3/2_ and Ni 2*p*_1/2_ severally, which were the characteristic peaks of the Ni^2+^^57,58^. The Ni 2*p* binding energy of NiRu_0.13_-BDC is higher than that of Ni-BDC, suggesting strong electron interaction between Ni and Ru atoms and electron depletion on Ni. The O 1*s* spectra in Fig. 2d, can be deconvoluted into three peaks with their binding energies at 531.3, 532.0 and 532.9 eV, assigned to the Ni(Ru)–O, O–C=O, and absorbed water species respectively^57^.

**Fig. 3:**
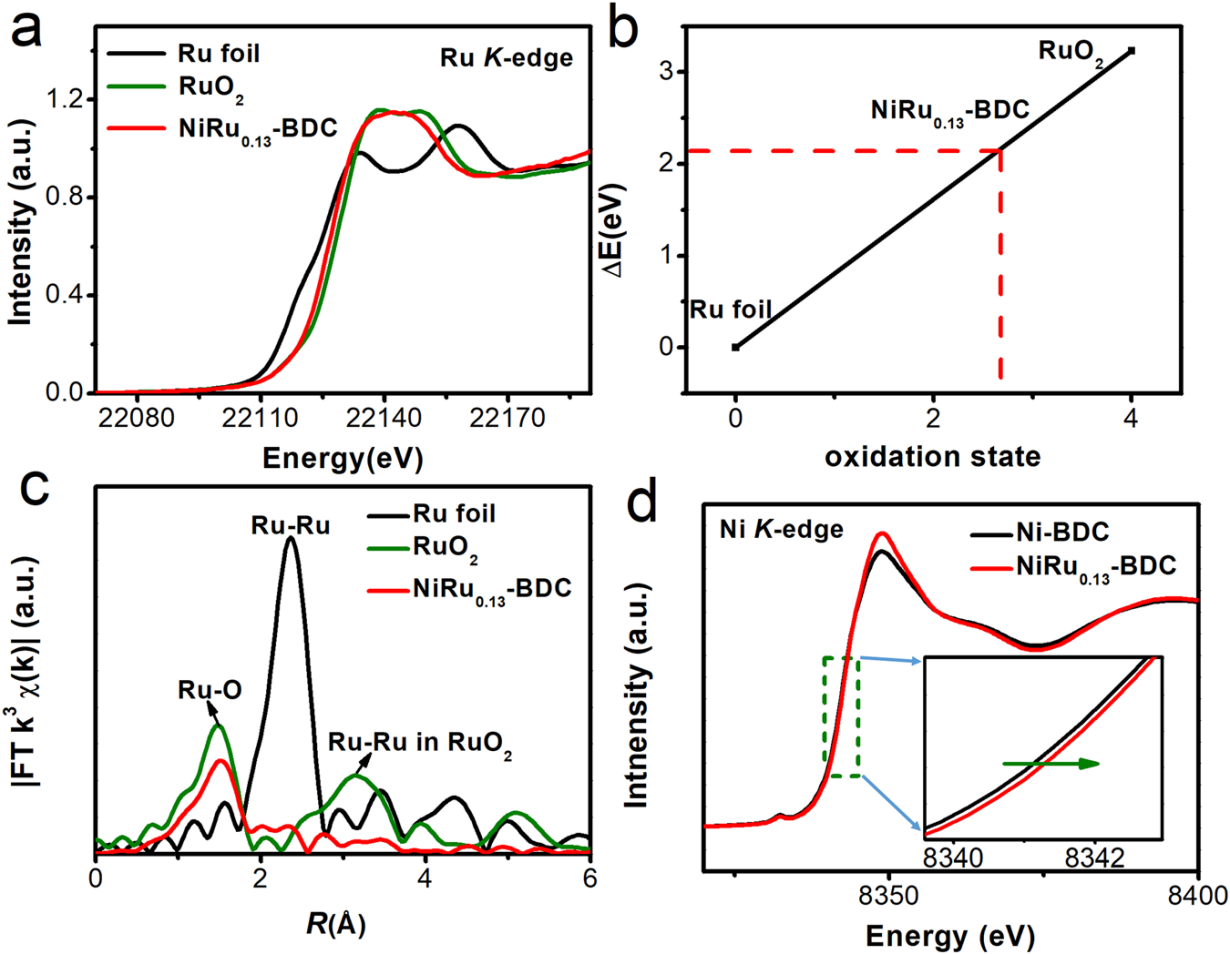
Electronic structure characterization of NiRu_0.13_-BDC. **a** Ru *K*-edge XANES spectra of NiRu_0.13_-BDC, Ru foil and RuO_2_. **b** Relationship between Ru *K*-edge threshold value (*E*_0_) and oxidation state for NiRu_0.13_-BDC and two reference materials. **c** Fourier transformed EXAFS spectra of Ru foil, RuO_2_ and NiRu_0.13_-BDC. **d** Ni *K*-edge XANES spectra of Ni-BDC and NiRu_0.13_-BDC.

The local electronic structure and coordination environment of Ru in NiRu_0.13_-BDC were further investigated by X-ray absorption measurements. Figure 3a and Supplementary Fig. 7 show the X-ray absorption near-edge structure (XANES) of RuO_2_, Ru foil and NiRu_0.13_-BDC, it can be observed that the threshold value (*E*_0_) of the NiRu_0.13_-BDC (22129.17 eV) is between RuO_2_ (22130.25 eV) and Ru foil (22127.02 eV), indicating the valence state of Ru is between 0 and +4. In order to further estimate the valance state of the Ru atoms in NiRu_0.13_-BDC, we performed the ΔE_0_ as a function of oxidation state of Ru atoms in these materials^59^. Hence, the average valance state of Ru atoms in NiRu_0.13_-BDC is +2.67, as shown in Fig. 3b. The extended X-ray absorption fine structure (EXAFS) of NiRu_0.13_-BDC shows only a primary peak at 1.5 Å, which is assigned to Ru–O bond (Fig. 3c). Compared with Ru foil and RuO_2_, there are no obvious characteristic peaks can be identified to Ru–Ru metallic bond at 2.50 Å in Ru foil and Ru–Ru bond at 3.25 Å in RuO_2_^53^, revealing the Ru single-atom successfully dispersed in the Ni-BDC. In order to further confirm the structure of Ru in NiRu_0.13_-BDC, we fitted the Fourier transform XAFS in *R-*space of Ru *k*-edge using the structure model of replacing Ni atoms in Ni-BDC by Ru atoms. As can be seen from Supplementary Fig. 7c and Table 2, the fitting spectrum is well consistent with as measured, further confirming Ru single-atom replaced part of Ni in Ni-BDC. Taking account into XPS spectra and XRD pattern, Ru was atomically anchored in the MOF Ni-BDC. Furthermore, as shown in Fig. 3d and Supplementary Fig. 8, the energy of Ni positively shifted to higher in comparison with Ni-BDC, verifying the electron interaction between Ni and Ru atoms, which is in agreement with XPS analysis.

### Electrocatalytic performance toward HER

**Fig. 4:**
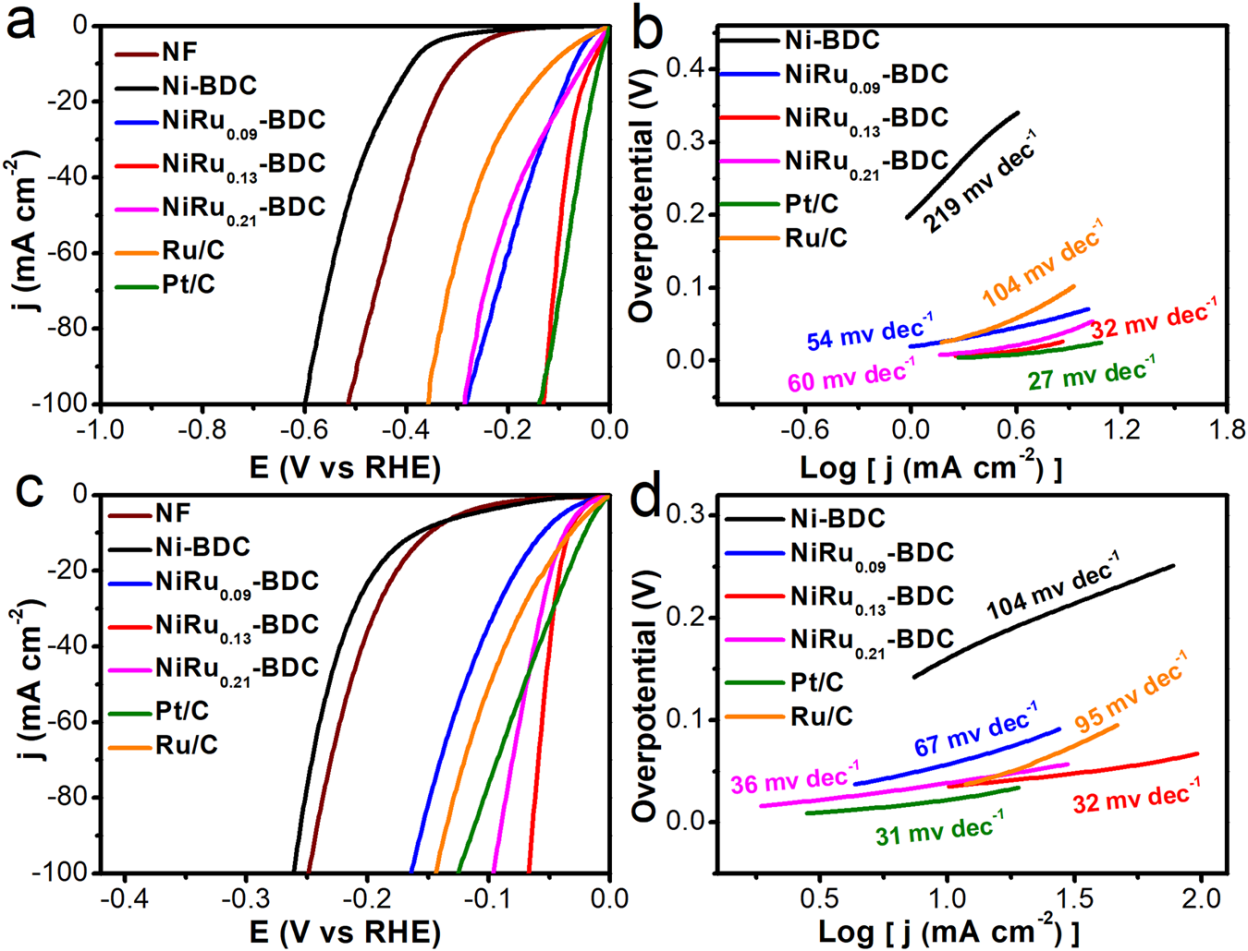
Electrochemical measurements. **a** LSV curves toward HER and **b** Tafel plots of Ni-BDC, NiRu_0.09_-BDC, NiRu_0.13_-BDC, NiRu_0.21_-BDC in 1 M PBS. **c** LSV curves toward HER and **d** Tafel plots of Ni-BDC, NiRu_0.09_-BDC, NiRu_0.13_-BDC, NiRu_0.21_-BDC in 1 M KOH.

The electrocatalytic performances of these catalysts were firstly measured in 1 M PBS solution with a three-electrode cell system under room temperature. And the loading mass of Ni-BDC, NiRu_0.09_-BDC, NiRu_0.13_-BDC, and NiRu_0.21_-BDC was about 2.5 mg cm^−2^. To evaluate the HER activity of these materials, commercial Pt/C and Ru/C were utilized as benchmarks with the same loading. As exhibited in Fig. 4a, Ni-BDC performed poor electrocatalytic activity with an overpotential of 389 mV to reach a current density of 10 mA cm^−2^. In contrast, after introducing Ru single-atom into Ni-BDC, the catalysts exhibited enhanced electrocatalytic performance toward HER. Remarkably, the NiRu_0.13_-BDC displayed high HER activity with a low overpotential of 36 mV at 10 mV cm^−2^ in 1 M PBS solution, which is much lower than that of Ru/C (115 mV) and even comparable to the commercial Pt/C (22 mV). Furthermore, NiRu_0.13_-BDC only needed an overpotential of 132 mV to reach a high current density of 100 mA cm^−2^, which was lower than that of Pt/C (139 mV) (Fig. 4a and Supplementary Fig. 9). The remarkable electroctatalytic performance of NiRu_0.13_-BDC also outperformed other previously reported HER electrocatalysts (Supplementary Table 3). Moreover, NiRu_0.21_-BDC with higher Ru content, displayed worse activity than NiRu_0.13_-BDC, further suggesting the superiority of single-atom NiRu_0.13_-BDC electrocatalyst. The Tafel slope (Fig. 4b) of NiRu_0.13_-BDC is 32 mV dec^−1^, lower than that of Ni-BDC (219 mV dec^−1^), NiRu_0.09_-BDC (54 mV dec^−1^) and NiRu_0.21_-BDC (60 mV dec^−1^), which is comparable to Pt/C, indicating that the NiRu_0.13_-BDC performed as the best catalyst of the series. The electrocatalytic performances were also tested in 1 M KOH and 1 M HCl. NiRu_0.13_-BDC also exhibited the best HER activity among these catalysts with an overpotential of 34 and 13 mV at a current density of 10 mA cm^−2^ in 1 M KOH and 1 M HCl, respectively, in Fig. 4c and Supplementary Fig. 9. The Tafel slope curves (Fig. 4d) also revealed NiRu_0.13_-BDC possessing smaller Tafel slope (32 mV dec^−1^) with accelerated HER kinetics in alkaline solution. The TOFs (Supplementary Fig. 10) of Ni-BDC, NiRu_0.09_-BDC, NiRu_0.13_-BDC, and NiRu_0.21_-BDC are 0.000048, 0.0032, 0.0091, and 0.0035 s^−1^, respectively. As can be seen from Supplementary Fig. 10, NiRu_0.13_-BDC has higher TOF value, indicating higher intrinsic activity. In addition, from the Supplementary Fig. 11, the faradic efficiency of NiRu_0.13_-BDC catalyst for HER is estimated to be close to 100%, indicating that almost all electrons are utilized for producing hydrogen.

To figure out the origin of the enhanced activity of the NiRu_0.13_-BDC electrocatalyst, the double-layer capacitances (*C*_dl_) measurements were carried out to evaluate the electrochemical active surface areas (Supplementary Fig. 12). NiRu_0.13_-BDC had higher *C*_dl_ (1.39 F cm^−2^)^60^ than that of Ni-BDC (0.0021 F cm^−2^), NiRu_0.09_-BDC (0.2797 F cm^−2^) and NiRu_0.21_-BDC (0.7572 F cm^−2^), indicating more electroactive surface exposed in NiRu_0.13_-BDC compared with pure Ni-BDC. The electrochemical impedance spectroscopy (EIS) was performed to deeply study the charge-transfer mechanism and the resulted Nyquist plots were shown in Supplementary Fig. 13 and Table 4. Apparently, the NiRu_0.13_-BDC presented a smaller *R*_ct_ value than other electrocatalysts, implying faster charge transfer. The long endurable stability was measured by chronoamperometry with an overpotential of 50 mV (vs RHE) in 1 M PBS solution. NiRu_0.13_-BDC exhibited good stability with a negligible current decrease after 30 h test (Supplementary Fig. 13). After stability test for 10 h, the nanosheets morphology maintained well (Supplementary Fig. 14). The main phases are NiRu_0.13_-BDC, indicating that the crystal structure of MOF catalyst showed limited changes (Supplementary Fig. 15). The XPS spectra of NiRu_0.13_-BDC after electrocatalysis exhibited that the peak of Ru 3*p* also showed limited changes (Supplementary Fig. 16), indicating the main component is still NiRu_0.13_-BDC MOF catalyst. The limited changes in crystal structure and chemical environment of NiRu_0.13_-BDC after 10 h HER stability test indicated the MOF had good stability.

### Density functional theory (DFT) calculations

**Fig. 5:**
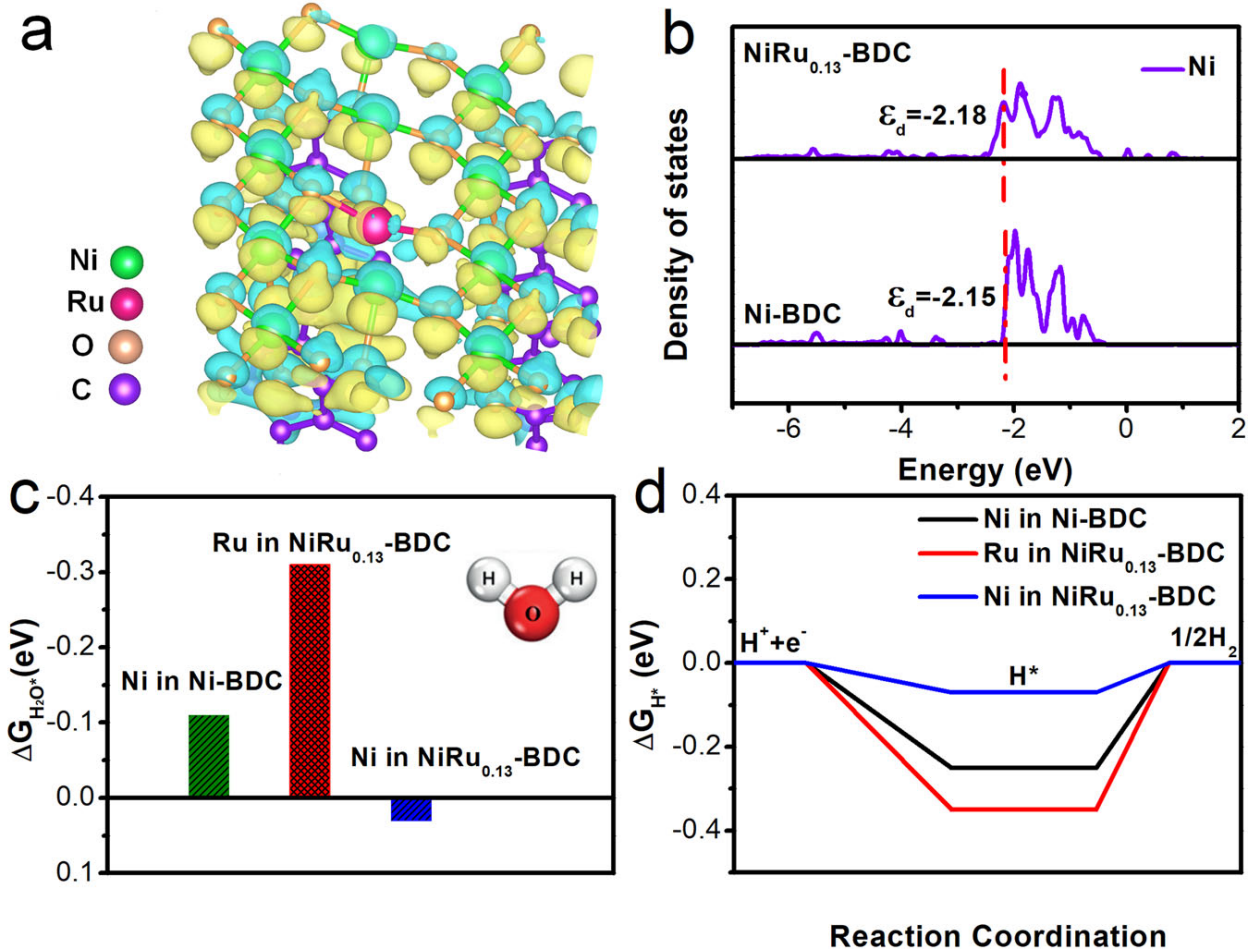
DFT calculations. **a** The charge density difference between Ni-BDC and NiRu_0.13_-BDC. The yellow and blue color represent charge accumulation and depletion, respectively. **b** Calculated DOS of Ni in Ni-BDC and NiRu_0.13_-BDC. **c** The calculated adsorption free energy of water on Ni-BDC and NiRu_0.13_-BDC. **d** Calculated free energy diagram of the HER.

To understand the effect of Ru single-atom in HER performance, we conducted DFT calculations. Supplementary Fig. 17 shows structure models of Ni-BDC and NiRu_0.13_-BDC (details in “Computation method”). For investigating the electronic structure of NiRu_0.13_-BDC, the charge density difference was firstly simulated. From Fig. 5a, there is overt charge accumulation around Ru and charge depletion around Ni, revealing charge interaction between Ni and Ru, which is in agreement with the above-mentioned XPS and XANES results. It further confirmed after introducing of Ru single-atom, the electronic structure of metal center was modulated. In order to further elucidate the origin of the improved HER activity of NiRu_0.13_-BDC after introducing Ru single-atom, the density of states (DOS) was also calculated to deeply investigate the changes of electronic structure of the catalyst. According to the partial and total DOS calculations (Fig. 5b and Supplementary Figs. 18, 19), with the formation of Ru single-atom in the MOF, the electronic structure of NiRu_0.13_-BDC changed. It should be noted that the d-band center of Ni shifted to lower energy (Fig. 5b), corresponding to a weaker H* adsorption on catalyst^61^. To elucidate the inherent relationship between the electronic structure and the enhanced electrocatalytic HER performance of NiRu_0.13_-BDC, the adsorption free energy of the HER intermediates was also calculated. Generally, there are two steps involved in neutral pH HER, including H_2_O adsorption/activation and H recombination on the surface of the catalyst^62^; so, a strong bonding of H_2_O and neither too strong nor too weak bonding of H to the surface are desired^63,64^. As displayed in Fig. 5c, Ru in NiRu_0.13_-BDC shows much lower adsorption energy of H_2_O (Δ*G*_H2O*_) compared with Ni in Ni-BDC and NiRu_0.13_-BDC, indicating the strongest water adsorption, which benefits for the following step to generate adsorbed H atoms^65,66^. In addition, the calculated Δ*G*_H*_ for adsorbed H atom forming molecular H_2_ is the key descriptor to predict and evaluate the activity for HER on catalyst surface^67^. While, Ru in NiRu_0.13_-BDC and Ni in Ni-BDC possess a more negative value of ΔG_H*_, indicating excessive strong adsorption of H and adverse for H desorption and H_2_ release. By contrast, the ΔG_H*_ of Ni in NiRu_0.13_-BDC (Fig. 5d) is closer to the optimal value of Δ*G*_H*_ = 0 eV, supporting high HER activity. For the NiRu_0.13_-BDC catalyst, with both Ni and Ru atoms in the crystal structure, it showcases a preferential HER activity. From the above results, it can be found that constructing Ru single-atom into the MOF can regulate the electronic states of Ni and Ru, and d-band center of Ni, resulting in an enhanced Δ*G*_H2O*_ and a more thermoneutral Δ*G*_H*_, thus devoting to improved HER performance.

## Discussion

In conclusion, we have synthesized the HER catalyst with significantly improved performance by introducing Ru single-atom into the MOF Ni-BDC. Impressingly, the as-sythesized NiRu_0.13_-BDC exhibits enhanced activity toward HER in all pH, comparable with commercial Pt/C, especially delivering a low overpotential of 36 mV at 10 mA cm^−2^ with a Tafel slope of 32 mV dec^−1^ in 1 M PBS solution. More improtantly, after introducing Ru, there is strong electron interaction between Ni and Ru atoms and electron depletion on Ni in the MOF from XPS results, also confirmed by EXAFS and XANES. DFT calculations disclose that the introduction of atomically dispersed Ru can promote the adsorption of H_2_O and optimize good thermoneutral ΔG_H*_ to facilitate H_2_ release on Ni site of the MOF by regulating electronic structure, thus effciently enhancing the HER activity. This work paves a way of designing efficient MOFs electrocatalysts via single-atom strategy to modulate electronic structure and intermediates asorption.

## Methods

### Chemicals

Ni(NO_3_)_2_·6H_2_O, RuCl_3_, Pt/C (20%), Ru/C (5%), *N*,*N*-Dimethylformami (DMF), ethanol, and terephthalic acid (H_2_BDC) were purchased from Aladdin (Shanghai, China). All the reagents were used without any further purification.

### Synthesis of Ni-BDC

The Ni-BDC was synthesized through a solvothermal method as following. First, the nickel foam was washed with 3 M HCl and water in the size of 1.5 × 3 cm^2^. Second, the as-processed nickel foam was immersed into a solution containing 4.5 mL DMF and 1 mmol Ni(NO_3_)_2_·6H_2_O in a Teflon-lined autoclave. Afterwords, a solution composed of 7.5 ml DMF, 1 mmol BDC and 1 ml 0.4 M NaOH was poured into the autoclave and subsequently heated at 100 °C for 15 h.

### Synthesis of NiRu_x_-BDC

The obtained Ni-BDC was immersed into an ethanol solution containing 50 mg RuCl_3_ in Teflon-lined autoclave and then heated at 80 °C for 12 h. Other samples were prepared in the similar way with an addition of RuCl_3_ of 20, 75 mg, respectively. And the as-obtained samples were named as NiRu_*x*_-BDC (*x* represents the molar ratio of Ru:Ni). The NiRu_0.13_-BDC powder sample was synthesized without addition of nickel foam with the same method.

### Characterization

Powder X-ray diffraction was conducted on Rigaku SmartLab diffractometer equipped with Cu Kα X-ray source (λ = 1.540598 Å). SEM measurements were operated on a Hitachi SU8010 system. TEM images were carried out on a JEM-1400Plus apparatus. Scanning transmission electron microscopy (STEM) and corresponding EDS mapping images were obtained from a JEOL JEM-ARM 200F equipped with energy dispersive X-ray spectrometer. X-ray photoelectron spectra were obtained from a Thermo fisher Scientific K-Alpha^+^ instrument. Inductively coupled plasma mass spectrometry (ICP-MS) was measured on Thermo Scientific iCAP RQ.

### Electrochemical measurements

The electrochemical measurements were evaluated in a standard three-electrode cell system by using a CHI 760D (Shanghai, China) instrument. The catalysts were cut into 0.5 × 1 cm^2^ pieces utilizing as the working electrodes. An Ag/AgCl (3 M KCl) electrode and carbon rod were used as the reference and counter electrode, respectively. The measured potential was converted relative to RHE according to the following equation: *E* (vs RHE) = *E*_Ag/AgCl_ + 0.21 + 0.059 × pH. The Linear sweep voltammetry (LSV) was conducted at a scan rate of 2 mV s^−1^ with the potential corrected for iR loss. The CV tests were studied in the potential range from 0.386 to 0.486 V (vs RHE) at different scan rates. By plotting the difference of current density (j) at 0.436 V (vs. RHE) against the scan rate, we gained a line where the slope is equal to the geometric double-layer capacitance (*C*_dl_). EIS was evaluated with 5 mV amplitude in a frequency range from 0.01 to 10,000 Hz at −1.036 V (vs. Ag/AgCl). The turnover frequency (s^−1^) can be estimated with the following equation: *TOF* = *I*/2*nF*; where *I* is the current (A) during LSV, *F* is the Faraday constant (96485.3 C mol^−1^), *n* is the number of active sites (mol). The factor 1/2 is based on the assumption that two electrons are necessary to form a hydrogen molecule. Faradic efficiency was evaluated in a H-type cell with an anion exchange membrane as the separator and a gas chromatography (SHIMADZU GC-2014) for the hydrogen gas detection.

### Computation method

All the calculations are performed in the framework of the density functional theory with the projector augmented plane-wave method, as implemented in the Vienna ab initio simulation package^68^. The generalized gradient approximation proposed by Perdew, Burke, and Ernzerhof is selected for the exchange-correlation potential^69^. The Ni-BDC crystal structure has been modeled using a single periodic slab with a (4 × 2) supercell based on the previously reported MOF^50^, and the (200) facet was investigated which was dominant facet according to the XRD patterns. The benzene ring was passivated with hydrogen. And the NiRu_0.13_-BDC structure was derived by replacing one Ni atom with Ru atom. The reasonable vacuum layers were set to 20 Å, in order to avoid the interaction between periodic structures. Furthermore, when a 30 Å vacuum layer was set, the energy deviation yields to be 0.1 meV/atom compared with that of 20 Å, for further calculations we chose the 20 Å vacuum layer. The cut-off energy for plane wave is set to 400 eV. The energy criterion is set to 10^−5^ eV in iterative solution of the Kohn–Sham equation. The Brillouin zone integration is performed using a 1 × 2 × 2 k-mesh, for the DOS calculations the 1 × 5 × 5 k-mesh was applied. The slab contains four layers with two layers are fixed. All the structures are relaxed until the residual forces on the atoms have declined to less than 0.05 eV/Å. The free energies are calculated by the formula: Δ*G* = Δ*E*_tot_ + Δ*E*_ZPE_ − *T*Δ*S*, where Δ*E*_tot_, Δ*E*_ZPE_, and Δ*S* are the changes in total energy of the system, vibrational zero-point energy, entropy during the reaction, respectively; while *T* represents temperature^70^.

## Supplementary information

Supplementary Information

## Data Availability

Full data supporting the findings of this study are available within the article and its Supplementary Information, as well as from the corresponding author upon reasonable request.
