# Supplementary material for: Modulating electronic structure of metal-organic frameworks by introducing atomically dispersed Ru for efficient hydrogen evolution
Source: Nat Commun. 2021 Mar 1;12:1369. doi: 10.1038/s41467-021-21595-5 (PMC7921655; doi:10.1038/s41467-021-21595-5)
Supplement: Supplementary file 1 — Supplementary Information [file 41467_2021_21595_MOESM1_ESM.pdf]

Supplementary Information

Modulating Electronic Structure of Metal-Organic Frameworks by Introducing Atomically Dispersed Ru for Efficient Hydrogen Evolution

Sun et al.

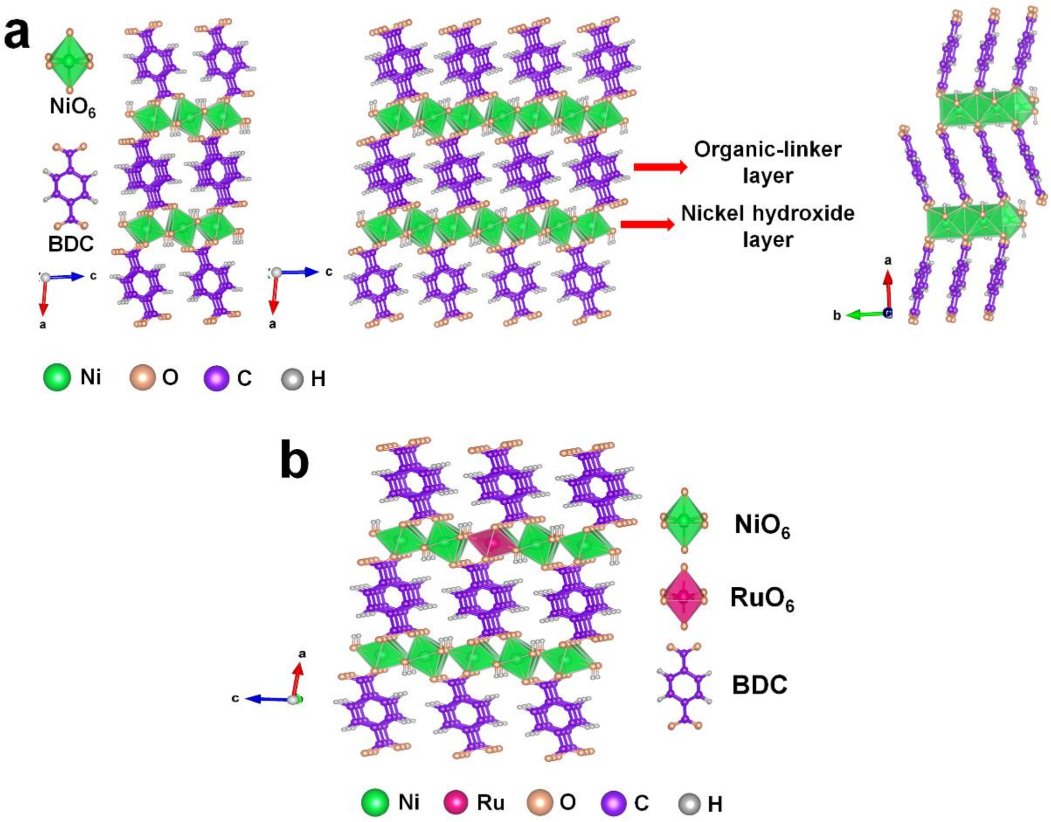

Supplementary Figure 1. Crystal structures. (a) crystal structure of $Ni_2(OH)_2(C_8H_4O_4)$ (named by Ni-BDC), (b) Crystal structure of $NiRu_{0.13}$-BDC derived from the known crystal structure of $Ni_2(OH)_2(C_8H_4O_4)$.

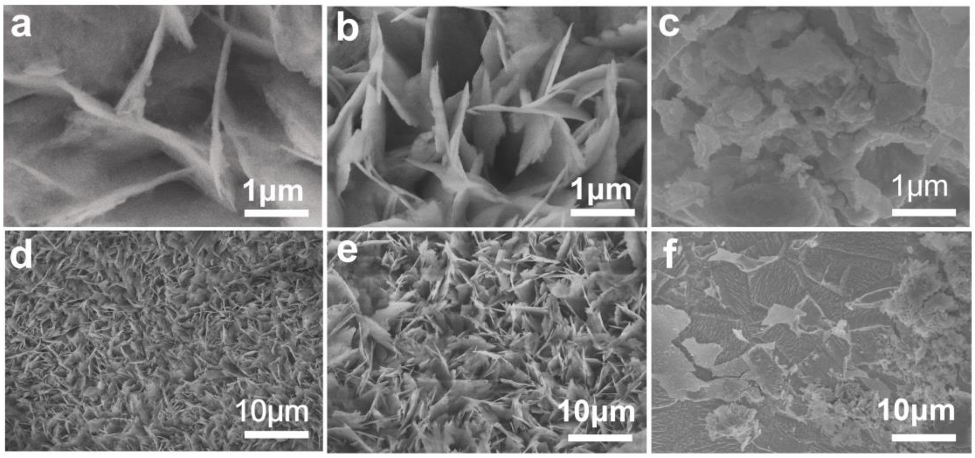

Supplementary Figure 2. SEM images of different catalysts. (a) and (d) Ni-BDC, (b) and (e) $NiRu_{0.09}$-BDC, (c) and f) $NiRu_{0.21}$-BDC .

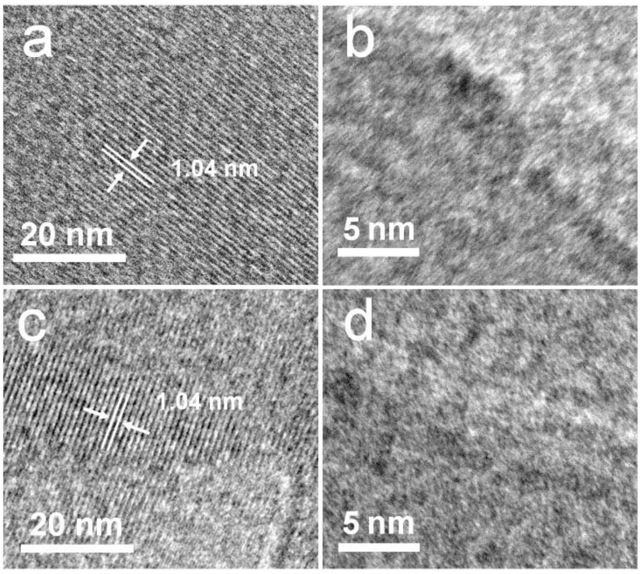

Supplementary Figure 3. HRTEM images of different catalysts. (a) and (b) $NiRu_{0.13}$-BDC, (c) and (d) Ni-BDC

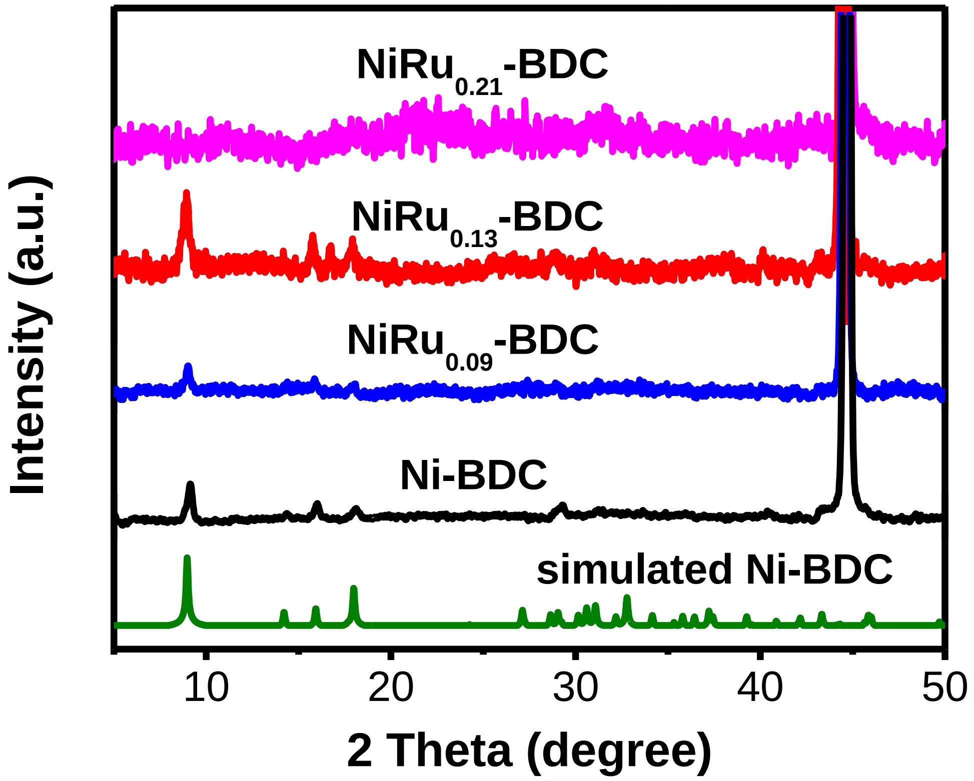

Supplementary Figure 4. XRD patterns of Ni-BDC, $NiRu_{0.09}$-BDC, $NiRu_{0.13}$-BDC and $NiRu_{0.21}$-BDC on nickel foam.

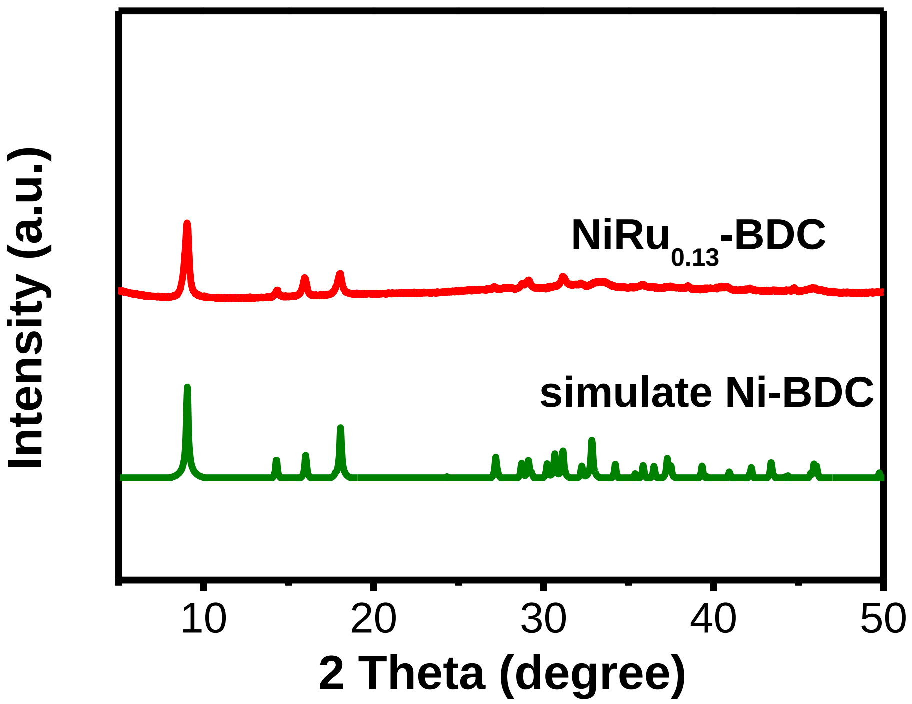

Supplementary Figure 5. XRD patterns of $NiRu_{0.13}$-BDC powder.

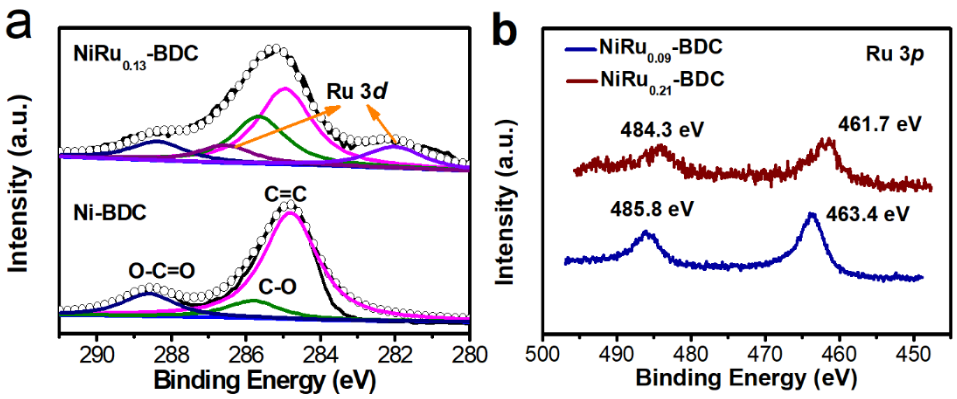

Supplementary Figure 6. XPS spectroscopic studies. (a) C 1*s* and Ru 3*d* spectra of Ni-BDC and $NiRu_{0.13}$-BDC. (b) Ru 3*p* spectra of $NiRu_{0.09}$-BDC and $NiRu_{0.21}$-BDC.

Supplementary Table 1. The molar ratio of Ni/Ru in $NiRu_x$-BDC.

Molar ratio(Ni:Ru)	$NiRu_{0.09}$-BDC	$NiRu_{0.13}$-BDC	$NiRu_{0.21}$-BDC
ICP-MS analysis	1:0.09	1:0.13	1:0.21

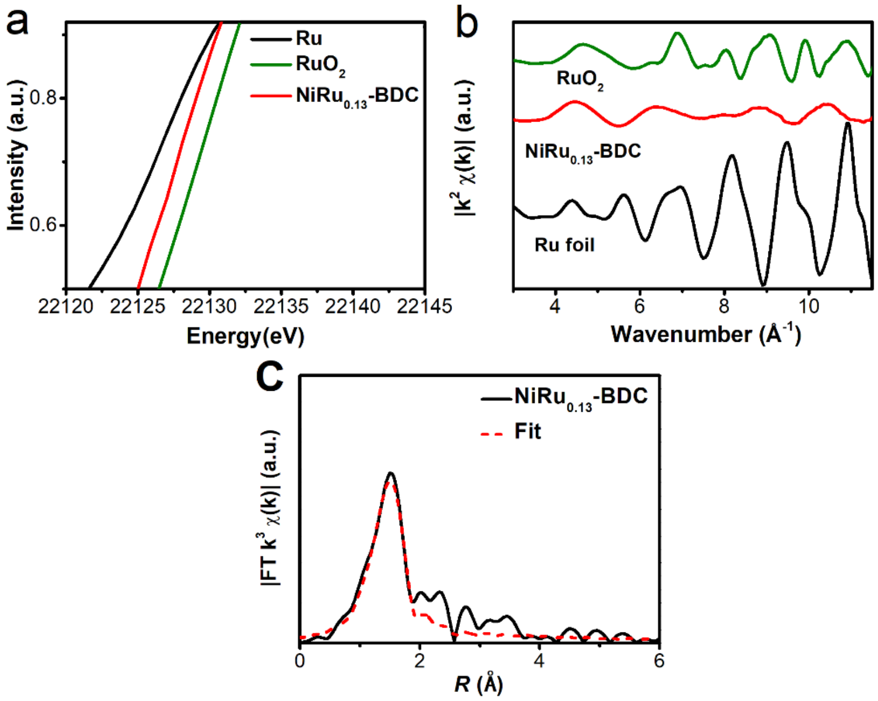

Supplementary Figure 7. X-ray absorption spectra. (a) The magnified Ru *K*-edge XANES spectra of $NiRu_{0.13}$-BDC, Ru foil and RuO_2, respectively. (b) Ru *K*-edge EXAFS oscillation functions $k^3\chi$ of $NiRu_{0.13}$-BDC, Ru foil, and RuO_2, respectively. c) EXAFS fitting curve for $NiRu_{0.13}$-BDC.

Supplementary Table 2. Fitting parameters of Ru K-edge EXAFS curves for $NiRu_{0.13}$-BDC.

Sample	Path	R(Å)[a]	N[b]	$\sigma^2(10^{-3}Å^2)$[c]
$NiRu_{0.13}$-BDC	Ru-O	2.09±0.02	6	7.59±3.7

[a] R: distance between absorber and backscatter atoms; [b] N: coordination number; [c] σ^2: Debye-Waller factor

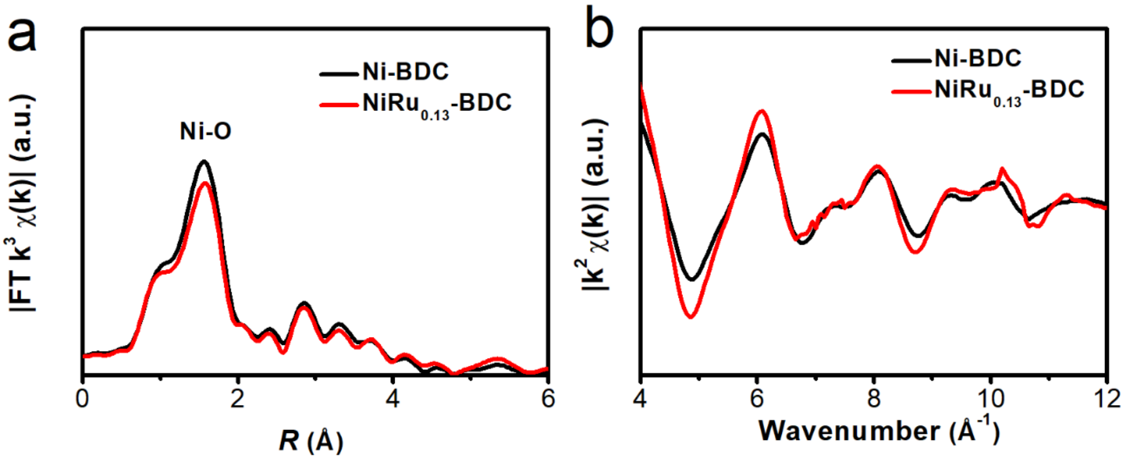

Supplementary Figure 8. X-ray absoption spectroscopic studies. (a) Ni *K*-edge Fourier transformed EXAFS spectra of Ni-BDC and $NiRu_{0.13}$-BDC. (b) Ni *K*-edge EXAFS oscillation functions $k^3\chi$ of Ni-BDC and $NiRu_{0.13}$-BDC, respectively.

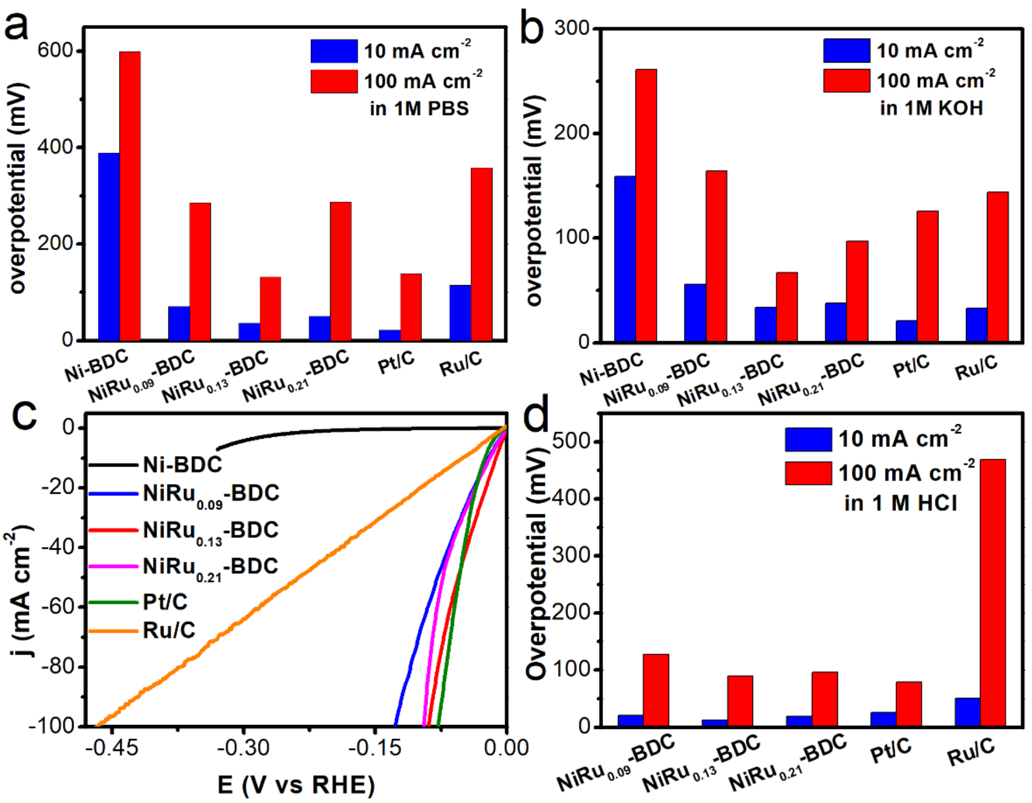

Supplementary Figure 9. HER performance. (a) The corresponding overpotentials in 1M PBS and (b) in 1 M KOH of Ni-BDC, $NiRu_{0.09}$-BDC, $NiRu_{0.13}$-BDC and $NiRu_{0.21}$-BDC at 10 mA cm^{-2} and 100 mA cm^{-2}. (c) LSV curves of Ni-BDC, $NiRu_{0.09}$-BDC, $NiRu_{0.13}$-BDC and $NiRu_{0.21}$-BDC toward HER in 1 M HCl. (d) The corresponding overpotentials of different catalysts in 1M HCl.

Supplementary Table 3. Comparisons of HER activity for $NiRu_{0.13}$ and other reported state of art elelctrocatalysis.

Catalyst	Media	Overpotential at 10 mA cm^{-2} (mV)	Substrate	Reference
	1 M PBS	36	Nickel foam	**This work**
$NiRu_{0.13}$-BDC	1 M KOH	34	Nickel foam	**This work**
	1 M HCl	13	Nickel foam	**This work**
karst NF	1 M PBS	60	Nickel foam	1
CrO_x/Cu–Ni	1 M PBS	48	Cu foam	2
$Ni_{0.89}Co_{0.11}Se_2$ MNSN/ NF	1 M PBS	82	Nickel foam	3
N–Co_2P/CC	1 M PBS	42	Carbon cloth	4
$NiCo_2P_x$/CF	1 M PBS	63	Carbon fiber	5
Mn-CoP/Ti	1 M PBS	86	Ti mesh	6
PtSA-NT-NF	1 M PBS	24	Nickel foam	7
N-Ni	1 M PBS	64	Nickel foam	8
N,Mn-MoS_2/NF	1 M PBS	70	Nickel foam	9
NiFeRu-LDH	1 M KOH	29	nickel foam	10
Ni, Zn dual-doped CoO NRs	1 M KOH	53	CFP	11
R-MoS_2/NF	1 M KOH	71	nickel foam	12
$CoSe_2$	1 M KOH	79	nickel foam	13
Ni−$N_{0.19}$	1 M KOH	42	CFP	14
Ni_3N/Ni foam	1 M KOH	100	nickel foam	15
V_8C_7	0.5 M H_2SO_4	38	nickel foam	16
CoMoNiS-NF-31	0.5 M H_2SO_4	103	nickel foam	17
FLNPC@MoP-NC/MoP-C	0.5 M H_2SO_4	74	Carbon Fiber	18
Mo_2C-MoOx/CC(	1 M $HClO_4$	74	Carbon cloth	19
Fe-doped CoP/Ti	0.5 M H_2SO_4	78	Ti mesh	20

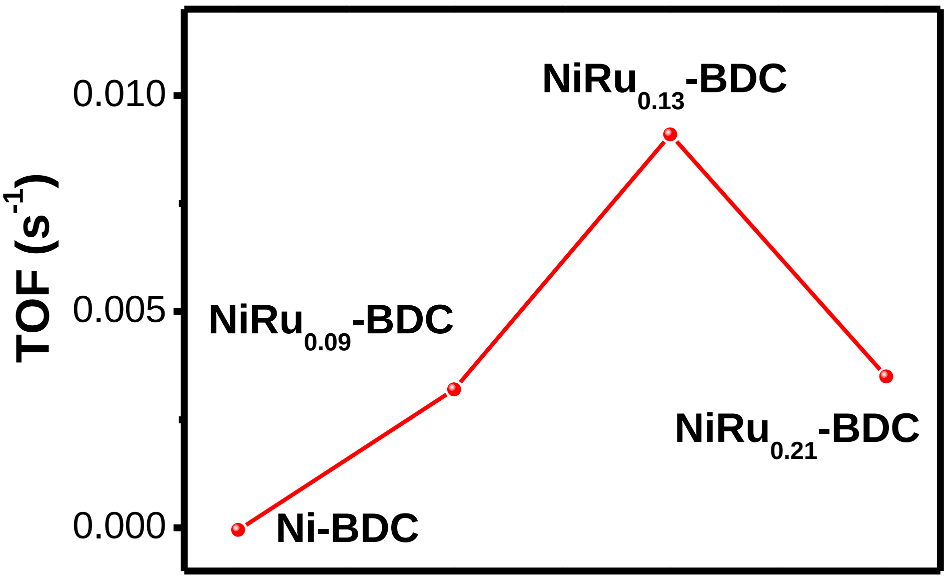

Supplementary Figure 10. Turnover frequency (TOF) of different materials at an overpotential of 100 mV.

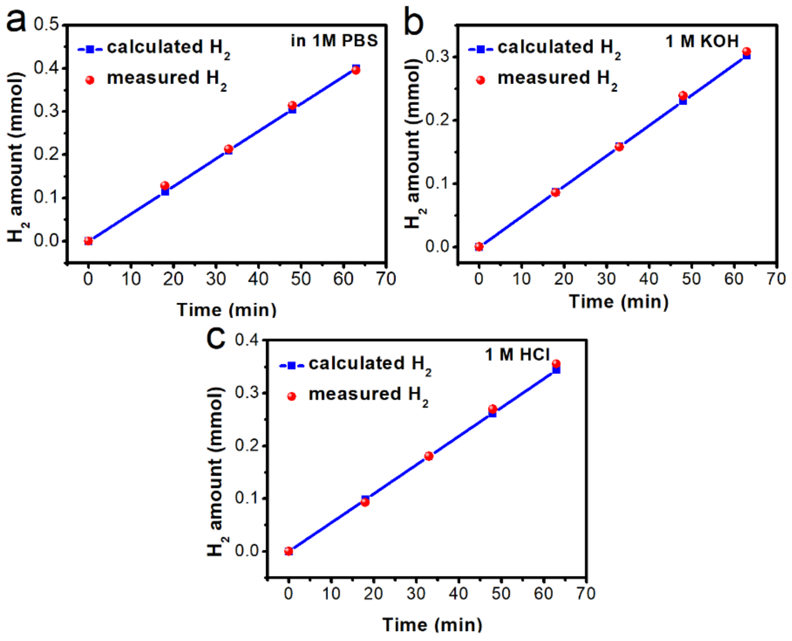

Supplementary Figure 11. Faradic efficiency measurements. The amount of theoretically calculated and experimentally measured hydrogen at different reaction times on $NiRu_{0.13}$-BDC with a current density of 20.5, 15.5, 17.6 mA in 1 M PBS (a), 1 M KOH (b) and 1 M HCl (c), respectively.

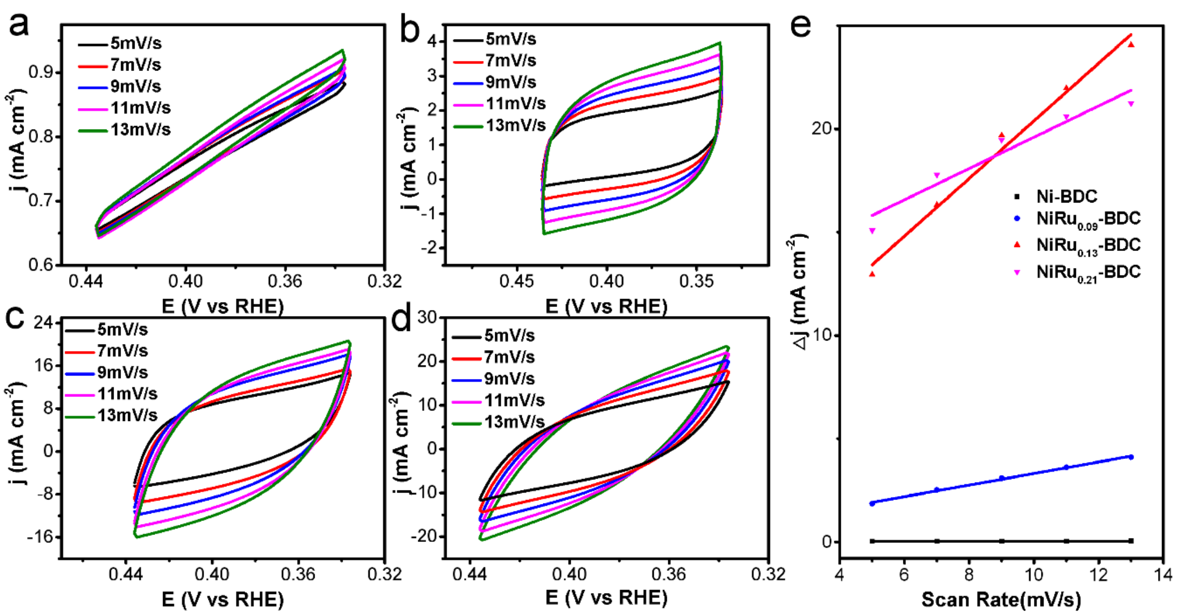

Supplementary Figure 12. CV plots and double-layer capacitance of different catalysts. CV plots of (a) Ni-BDC, (b) $NiRu_{0.09}$-BDC, (c) $NiRu_{0.13}$-BDC and (d) $NiRu_{0.21}$-BDC at different scan rates. (e) Double-layer capacitance of Ni-BDC, $NiRu_{0.09}$-BDC, $NiRu_{0.13}$-BDC and $NiRu_{0.21}$-BDC.

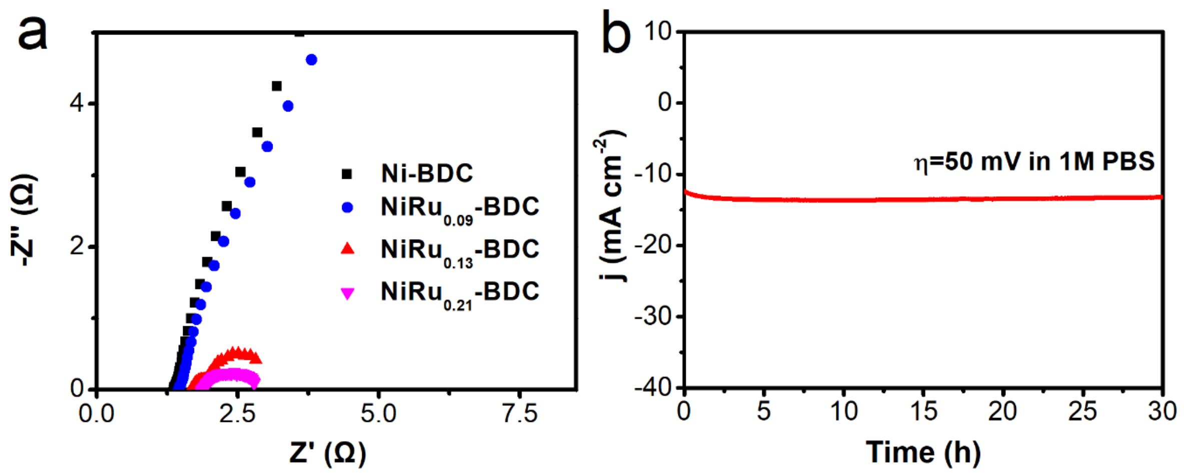

Supplementary Figure 13. Electrochemical performance of $NiRu_{0.13}$-BDC. (a) Electrochemical impedance spectra of different catalysts, (b) chronoamperometry curves of $NiRu_{0.13}$-BDC in 1 M PBS.

Supplementary Table 4. EIS results of Ni-BDC, $NiRu_{0.09}$-BDC, $NiRu_{0.13}$-BDC and $NiRu_{0.21}$-BDC

Catalyst	Solution series resistances R_s (Ω)	Charge transfer resistance R_{ct} (Ω)
Ni-BDC	1.42	19.56
$NiRu_{0.09}$-BDC	1.45	11.41
$NiRu_{0.13}$-BDC	1.68	0.31
$NiRu_{0.21}$-BDC	1.67	0.66

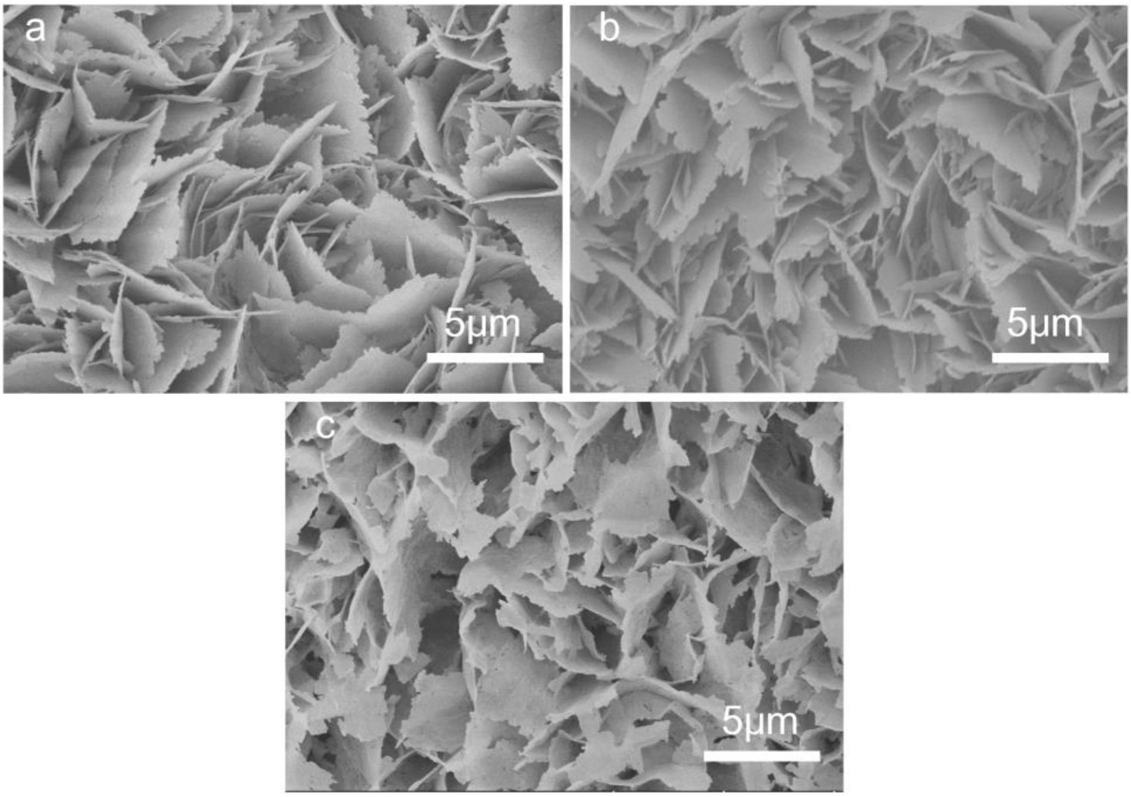

Supplementary Figure 14. SEM images of $NiRu_{0.13}$-BDC afters stability test. (a) before HER, (b) (c) after 10h, 30h electrocatalytic stability test at an overpotential of 50 mV in 1 M PBS.

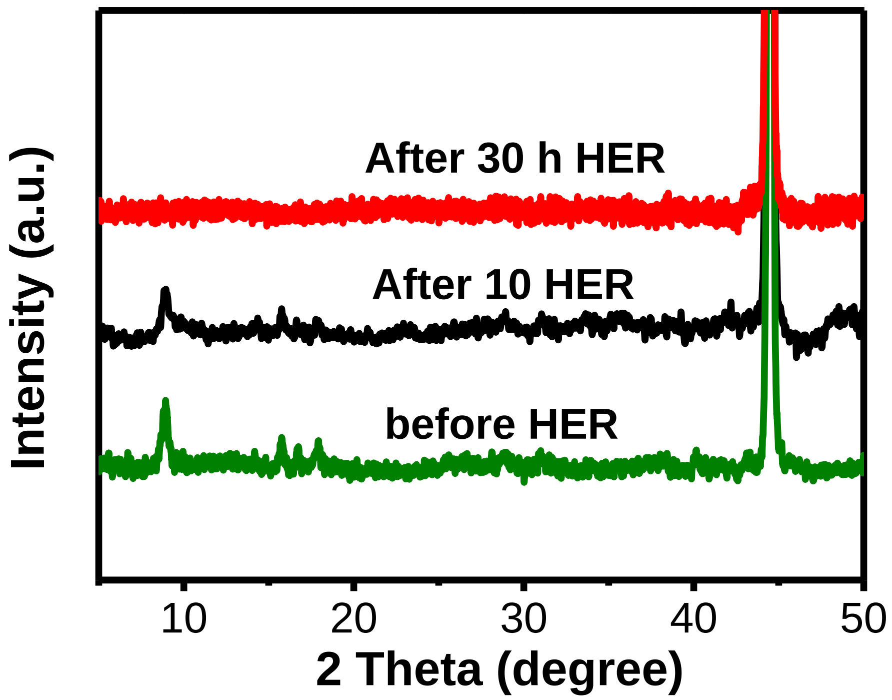

Supplementary Figure 15. XRD patterns of $NiRu_{0.13}$-BDC before HER, after 10h, 30h electrocatalytic stability test at an overpotential of 50 mV in 1 M PBS.

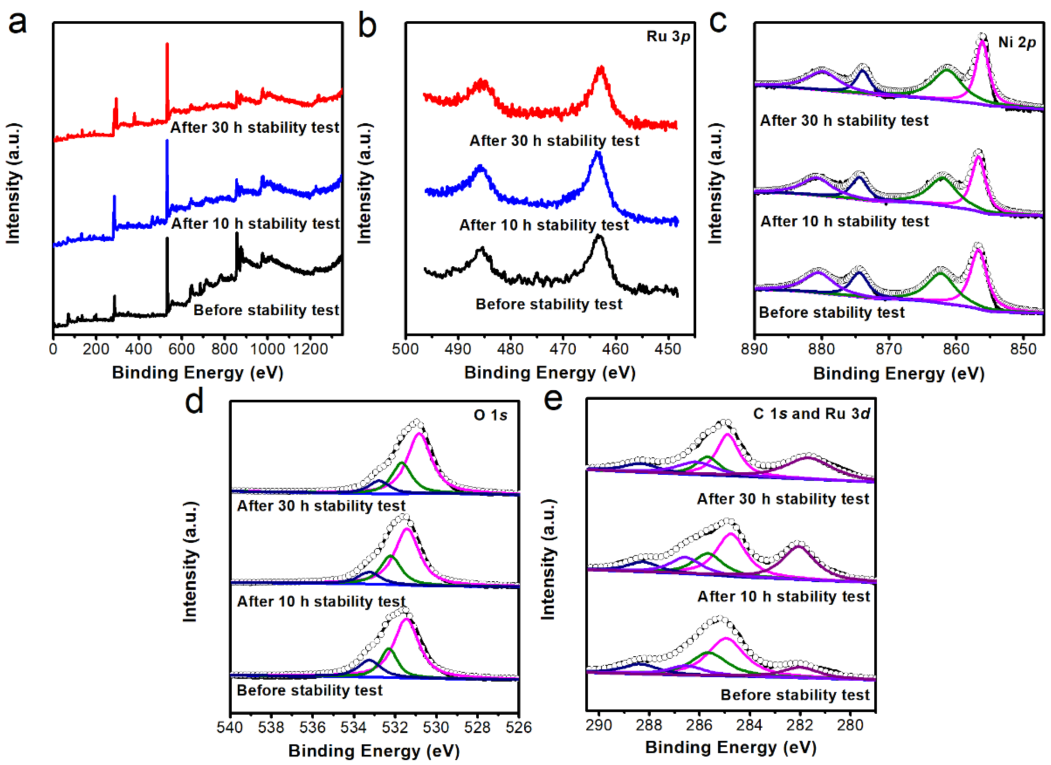

Supplementary Figure 16. XPS spectra of $NiRu_{0.13}$-BDC after stability test. (a) Full range XPS patterns, (b) Ru 3*p*, (c) Ni 2*p*, (d) O 1*s* and (e) C 1*s* and Ru 3*d* XPS spectra of $NiRu_{0.13}$-BDC before HER and after 10 h, 30 h electrocatalytic test.

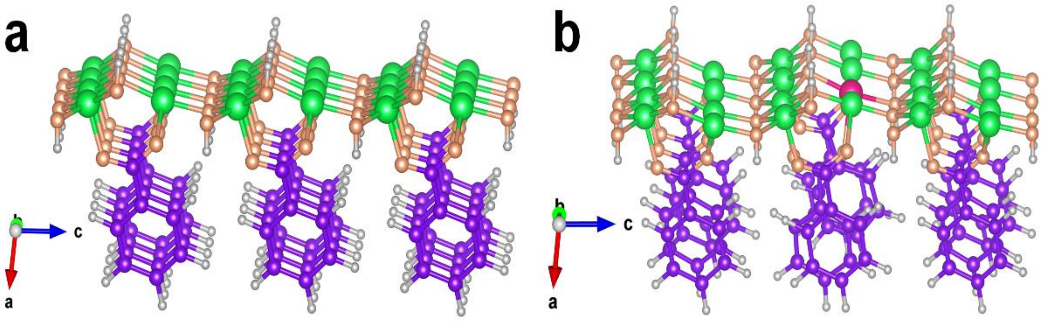

Supplementary Figure 17. Crystal structure models. (a) Ni-BDC, (b) $NiRu_{0.13}$-BDC.

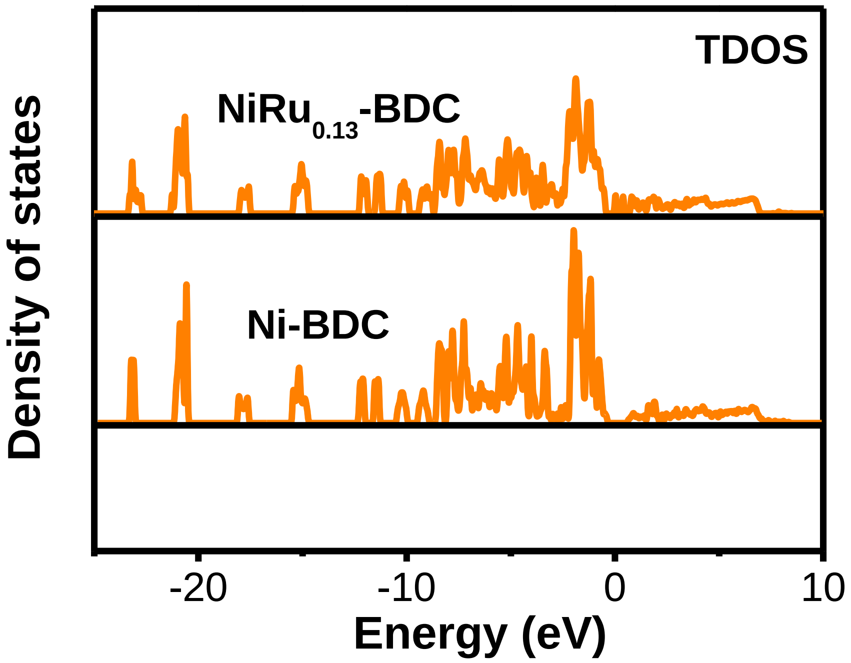

Supplementary Figure 18. Calculated total DOS of Ni-BDC and $NiRu_{0.13}$-BDC.

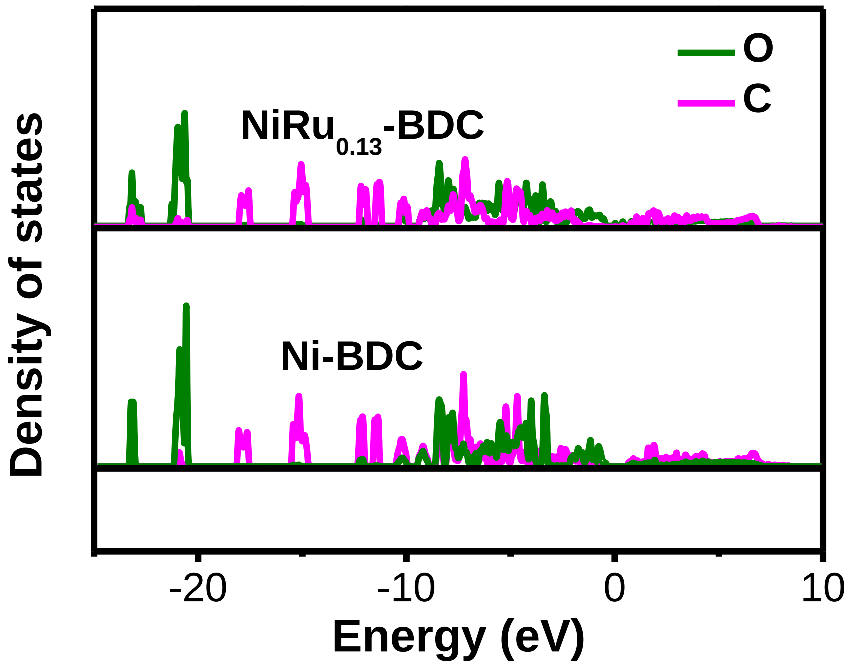

Supplementary Figure 19. Calculated partial density of states (PDOS) of Ni-BDC and $NiRu_{0.13}$-BDC.

References

1. Gao, X. *et al.* Karst landform-featured monolithic electrode for water electrolysis in neutral media. *Energy Environ. Sci.* **13**, 174-182 (2020).
2. Dinh, C.-T. *et al.* Multi-site electrocatalysts for hydrogen evolution in neutral media by destabilization of water molecules. *Nat. Energy* **4**, 107-114 (2018).
3. Liu, B. *et al.* Nickel-Cobalt Diselenide 3D Mesoporous Nanosheet Networks Supported on Ni Foam: An All-pH Highly Efficient Integrated Electrocatalyst for Hydrogen Evolution. *Adv. Mater.* **29**, 1606521 (2017).
4. Men, Y. *et al.* Tailoring the Electronic Structure of Co_2P by N Doping for Boosting Hydrogen Evolution Reaction at All pH Values. *ACS Catal.* **9**, 3744-3752 (2019).
5. Zhang, R. *et al.* Ternary $NiCo_2P_x$ Nanowires as pH-Universal Electrocatalysts for Highly Efficient Hydrogen Evolution Reaction. *Adv. Mater.* **29**, 1605502 (2017).
6. Liu, T. *et al.* Mn Doping of CoP Nanosheets Array: An Efficient Electrocatalyst for Hydrogen Evolution Reaction with Enhanced Activity at All pH Values. *ACS Catal.* **7**, 98-102 (2016).
7. Zhang, L., Han, L., Liu, H., Liu, X. & Luo, J. Potential-Cycling Synthesis of Single Platinum Atoms for Efficient Hydrogen Evolution in Neutral Media. *Angew. Chem. Int. Ed.* **56**, 13694-13698 (2017).
8. You, B. *et al.* Universal Surface Engineering of Transition Metals for Superior Electrocatalytic Hydrogen Evolution in Neutral Water. *J. Am. Chem. Soc.* **139**, 12283-12290 (2017).
9. Sun, T. *et al.* Engineering the Electronic Structure of MoS_2 Nanorods by N and Mn Dopants for Ultra-Efficient Hydrogen Production. *ACS Catal.* **8**, 7585-7592 (2018).
10. Chen, G. *et al.* Accelerated Hydrogen Evolution Kinetics on NiFe-Layered Double Hydroxide Electrocatalysts by Tailoring Water Dissociation Active Sites. *Adv. Mater.* **30**, 1706279 (2018).
11. Ling, T. *et al.* Well-Dispersed Nickel- and Zinc-Tailored Electronic Structure of a Transition Metal Oxide for Highly Active Alkaline Hydrogen Evolution Reaction. *Adv. Mater.* **31**, 1807771 (2019).
12. Anjum, M. A. R., Jeong, H. Y., Lee, M. H., Shin, H. S. & Lee, J. S. Efficient Hydrogen Evolution Reaction Catalysis in Alkaline Media by All-in-One MoS_2 with Multifunctional Active Sites. *Adv. Mater.* **30**, 1707105 (2018).
13. Zhang, J. Y. *et al.* Anodic Hydrazine Oxidation Assists Energy-Efficient Hydrogen Evolution over a Bifunctional Cobalt Perselenide Nanosheet Electrode. *Angew. Chem. Int. Ed.* **57**, 7649-7653 (2018).
14. Li, Y. *et al.* Processable Surface Modification of Nickel-Heteroatom (N, S) Bridge Sites for Promoted Alkaline Hydrogen Evolution. *Angew. Chem. Int. Ed.* **58**, 461-466 (2019).

15. Ledendecker, M., Schlott, H., Antonietti, M., Meyer, B. & Shalom, M. Experimental and Theoretical Assessment of Ni-Based Binary Compounds for the Hydrogen Evolution Reaction. *Adv. Energy Mater.* **7**, 1601735 (2017).
16. Xu, H. *et al.* A New Platinum-Like Efficient Electrocatalyst for Hydrogen Evolution Reaction at All pH: Single-Crystal Metallic Interweaved V_8C_7 Networks. *Adv. Energy Mater.* **8**, 1800575 (2018).
17. Yang, Y. *et al.* Hierarchical Nanoassembly of $MoS_2/Co_9S_8/Ni_3S_2/Ni$ as a Highly Efficient Electrocatalyst for Overall Water Splitting in a Wide pH Range. *J. Am. Chem. Soc.* **141**, 10417-10430 (2019).
18. Liu, B. *et al.* Few Layered N, P Dual-Doped Carbon-Encapsulated Ultrafine MoP Nanocrystal/MoP Cluster Hybrids on Carbon Cloth: An Ultrahigh Active and Durable 3D Self-Supported Integrated Electrode for Hydrogen Evolution Reaction in a Wide pH Range. *Adv. Funct. Mater.* **28**, 1801527 (2018).
19. He, L. *et al.* Molybdenum Carbide-Oxide Heterostructures: In Situ Surface Reconfiguration toward Efficient Electrocatalytic Hydrogen Evolution. *Angew. Chem. Int. Ed.* **59**, 3544-3548 (2020).
20. Tang, C. *et al.* Fe-Doped CoP Nanoarray: A Monolithic Multifunctional Catalyst for Highly Efficient Hydrogen Generation. *Adv. Mater.* **29**, 1602441 (2017).
